# Pancracine, a Montanine-Type Amaryllidaceae Alkaloid, Inhibits Proliferation of A549 Lung Adenocarcinoma Cells and Induces Apoptotic Cell Death in MOLT-4 Leukemic Cells

**DOI:** 10.3390/ijms22137014

**Published:** 2021-06-29

**Authors:** Darja Koutová, Radim Havelek, Eva Peterová, Darina Muthná, Karel Královec, Kateřina Breiterová, Lucie Cahlíková, Martina Řezáčová

**Affiliations:** 1 Department of Medical Biochemistry, Faculty of Medicine in Hradec Kralove, Charles University, Simkova 870, 500 03 Hradec Kralove, Czech Republic; koutova.darja@lfhk.cuni.cz (D.K.); PETEROVE@lfhk.cuni.cz (E.P.); MuthnaD@lfhk.cuni.cz (D.M.); rezacovam@lfhk.cuni.cz (M.Ř.); 2 Department of Biological and Biochemical Sciences, Faculty of Chemical Technology, University of Pardubice, Studentska 573, 532 10 Pardubice, Czech Republic; karel.kralovec@upce.cz; 3 ADINACO Research Group, Department of Pharmaceutical Botany, Faculty of Pharmacy, Charles University, Heyrovskeho 1203, 500 05 Hradec Kralove, Czech Republic; breiterk@faf.cuni.cz (K.B.); cahlikova@faf.cuni.cz (L.C.)

**Keywords:** Amaryllidaceae alkaloids, pancracine, cytotoxicity, antiproliferative activity, cell cycle arrest, apoptosis

## Abstract

Pancracine, a montanine-type Amaryllidaceae alkaloid (AA), is one of the most potent compounds among natural isoquinolines. In previous studies, pancracine exhibited cytotoxic activity against diverse human cancer cell lines in vitro. However, further insight into the molecular mechanisms that underlie the cytotoxic effect of pancracine have not been reported and remain unknown. To fill this void, the cell proliferation and viability of cancer cells was explored using the Trypan Blue assay or by using the xCELLigence system. The impact on the cell cycle was determined by flow cytometry. Apoptosis was evaluated by Annexin V/PI and by quantifying the activity of caspases (-3/7, -8, and -9). Proteins triggering growth arrest or apoptosis were detected by Western blotting. Pancracine has strong antiproliferative activity on A549 cells, lasting up to 96 h, and antiproliferative and cytotoxic effects on MOLT-4 cells. The apoptosis-inducing activity of pancracine in MOLT-4 cells was evidenced by the significantly higher activity of caspases. This was transmitted through the upregulation of p53 phosphorylated on Ser392, p38 MAPK phosphorylated on Thr180/Tyr182, and upregulation of p27. The pancracine treatment negatively altered the proliferation of A549 cells as a consequence of an increase in G1-phase accumulation, associated with the downregulation of Rb phosphorylated on Ser807/811 and with the concomitant upregulation of p27 and downregulation of Akt phosphorylated on Thr308. This was the first study to glean a deeper mechanistic understanding of pancracine activity in vitro. Perturbation of the cell cycle and induction of apoptotic cell death were considered key mechanisms of pancracine action.

## 1. Introduction

Alkaloids are a large group of important natural compounds, and, thanks to their prophylactic or therapeutic values, they are undoubtedly a stable player in the field of medical treatment for many diseases. Approximately 50–60% of the new drugs approved between 1981 and 2010 either originated from plants or were prepared by variations of the chemical structures of the compounds previously described in plants [1]. Alkaloids isolated from Amaryllidaceae plants comprise a large group of approximately 600 naturally occurring isoquinoline alkaloids with a vast structural diversity [2]. Therefore, the large number of Amaryllidaceae alkaloids (AA) have been divided into several structurally divergent skeleton types according to their biosynthetic origin, and ring structure and named after a representative alkaloid from the class [2,3].

**Figure 1:**
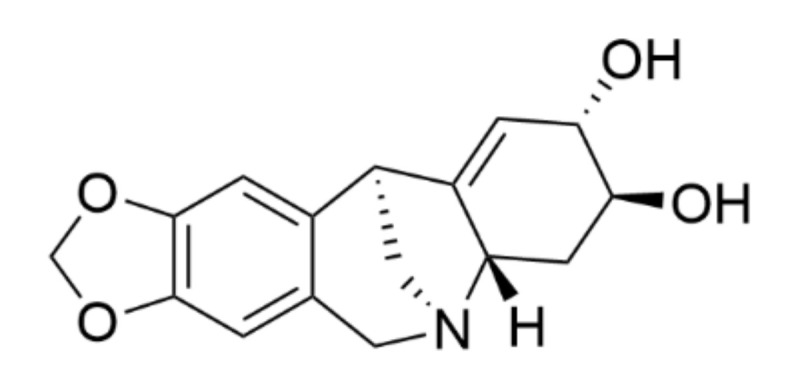
Chemical structure of pancracine.

Pancracine (Figure 1) belongs to the montanine types of AA, which possess a unique pentacyclic 5,11-methanomorphanthridine skeleton ring. At present, fourteen known AA share the 5,11-methanomorphanthridine scaffold, which is believed to be a significant carrier of the biological effects of montanine-type alkaloids [4,5]. Pancracine is distinct from other alkaloids of montanine types because of the presence of a double bond between C1 and C11a and two hydroxyls on stereocenters at C2 and C3 in the E ring of the 5,11-methanomorphanthridine framework [6]. Among the montanine skeleton type, montanine, coccinine, manthidine, and manthine were the first alkaloid members isolated from the *Haemanthus* species of the Amaryllidaceae plant family by Wildman et al. [7]. Thirteen years later, in 1968, Wildman and coworkers described another AA of a montanine type, pancracine, which they isolated from *Pancratium maritimum*, *Narcissus poeticus*, and *Rhodophiala bifida* [4]. Since then, attention has turned to investigations performed to gain a deeper understanding of montanine-type alkaloids, including either the possibility of isolation from other plants of the Amaryllidaceae family [6], preparing them by chemical de novo synthesis [5], or exploring their biological activity [6,8,9,10].

In our previous work, we screened the cytotoxicity of AA of various structural types isolated from bulbs of *Narcissus* cv. Professor Einstein against a panel of eight cancer cell lines [6]. Among them, pancracine has shown the ability to reduce the proliferation of cancer cells of different histotypes with IC_50_ values reaching into the micromolar range after 48 h of treatment. MOLT-4, A549, HT-29, MCF-7, and SAOS-2 are sensitive cell lines with IC_50_ values below 3 μM, whereas Jurkat A2780 and HeLa are more resistant, with IC_50_ values between 3 and 6 μM [9]. Consistently, other structure–activity studies investigating the antiproliferative effect of pancracine isolated from *Pancratium canariense* described its strong inhibitory effect from 4.3 ± 0.7 µM to 9.1 ± 1.0 µM on the growth of four cancer cell lines derived from ovarian (A2780), lung (SW1573), breast (T47-D), and colon (WiDr) carcinoma [9]. Moreover, a recent study reported the preparation of semisynthetic montanine-type alkaloid derivatives, which were screened in vitro for their antiproliferative activities against a panel of six cancer cell lines. Although the parent alkaloid of the montanine-type, manthine, was the most effective, the C2-OH and C2-indole-substituted 5,11-methanomorphanthridine derivatives also showed good antiproliferative activity [5]. In what concerns the other bioactivities, there were two studies reporting the antibiotic, antifungal, and antiparasitic effects of pancracine [11,12]. Antimicrobial activity testing using an agar diffusion technique and bacteria species *Staphylococcus aureus*, *Escherichia coli*, and *Pseudomonas aeruginosa* and the yeast *Candida albicans* displayed good inhibitory activity of pancracine against *Staphylococcus aureus* and *Pseudomonas aeruginosa* and moderate activity against *Candida albicans* [11]. The interesting biological activity was complemented by a study describing the antiprotozoal activities of pancracine isolated from *Narcissus angustifolius* subsp. *transcarpathicus* against *Trypanosoma brucei rhodesiense*, *Trypanosoma cruzi*, and *Plasmodium falciparum* [12]. 

The AA and its derivatives exhibited various pharmacological activities. Most notably, the cytotoxic activity of the several AA were noteworthy [3,13,14]. Since recent studies have begun to uncover the mechanisms sustaining the cytotoxicity of AA, deeper investigations on some AA are still fragmentary. The initial pilot studies have suggested that the micromole cytotoxic potency of pancracine towards cancer cell lines deserves further in-depth investigation. Moreover, there is fundamental lack of reports on the mode of action of pancracine in vitro, greatly hindering further investigation on the montanine type of AA. The aim of this study was to reveal the mechanisms underlying the proliferative inhibition after pancracine treatment by evaluating its effect on cell cycle progression, apoptosis, and related signal transduction pathways.

## 2. Results

### 2.1. Pancracine Inhibits Proliferation of A549 Lung Cancer Cells and Reduces Both Proliferation and Viability of MOLT-4 Leukemic Cells

**Figure 2:**
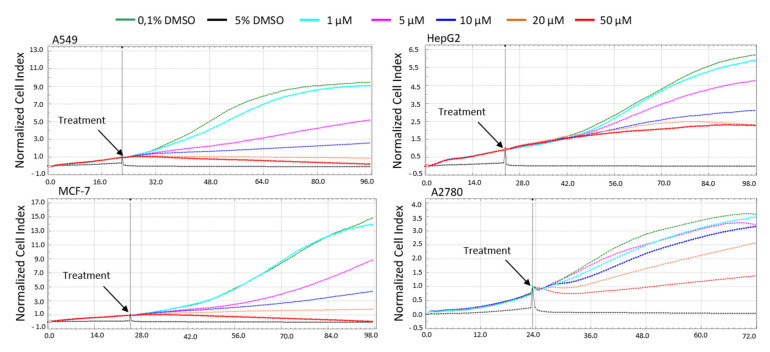
Dynamic real-time monitoring of proliferation and cytotoxicity using the xCELLigence system dedicated to adherent cancer cell lines. Growth kinetics are shown of human A549 lung adenocarcinoma, MCF-7 breast adenocarcinoma, HepG2 hepatocellular carcinoma, and A2780 ovarian carcinoma treated with pancracine. Cells treated with 0.1% DMSO were used as the vehicle control and 5% DMSO-treated cells as a positive control. The normalized cell index was measured over 72 h. Plots shown are representative of at least three replicate experiments in each case.

**Figure 3:**
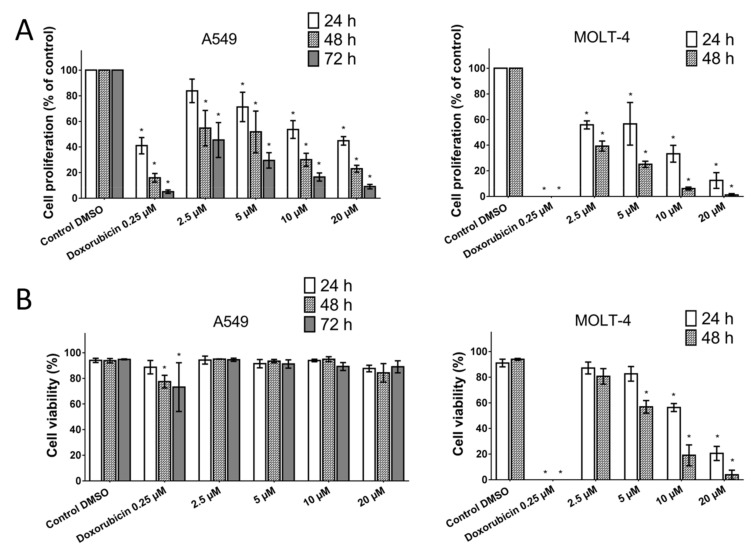
The effect of pancracine on the proliferation (**A**) and viability (**B**) of MOLT-4 and A549 cells. Changes in the proliferation and viability following pancracine treatment were monitored by the Trypan blue exclusion test at 24 and 48 h in MOLT-4 cells and at 24, 48, and 72 h in A549 cells. Results are shown as the mean ± SD from three experiments. * Significantly different to the control (*p* ≤ 0.05). Cells treated with 0.25-µM doxorubicin were used as a positive control.

The cytotoxicity of pancracine against a panel of cancer cell lines having different histotypes was previously described by our group in the study of Breiterová et al. [6], where the cytotoxicity of pancracine expressed as 50% inhibitory concentration (IC_50_) values was 2.29 ± 0.43 µM for A549 cells and 2.71 ± 0.25 µM for MOLT-4 cells. Following this study, which determined the cytotoxicity using the reduction of tetrazolium salt WST-1 as an end-point method, we decided to use the real-time label-free cell proliferation system xCELLigence RTCA. The xCELLigence system measures cell adhesion, viability, morphology, and the number of cells based on impedance, which are displayed as normalized cell index (CI) values. This system affords the continuous monitoring of cellular responses throughout an experiment, without the use of exogenous labels. Growth was observed in human A549 lung adenocarcinoma, MCF-7 breast adenocarcinoma, HepG2 hepatocellular carcinoma, and A2780 ovarian carcinoma treated with a range from 1 to 50 μM of pancracine, usually from 24 h after seeding, when the cells were in logarithmic growth and were treated, up to 96 h of incubation. Cells treated with 0.1% DMSO were used as a vehicle control and 5% DMSO-treated cells as a positive control. It is clear from the graph (Figure 2) that, with increasing the concentration of pancracine, there was a reduction in proliferation in all the tested cell lines. In the case of the A549 cell line, the treatment with 20 µM of pancracine stopped proliferation over the entire assay interval up to 96 h. The proliferation ability of A549 cells was also significantly reduced by 5 and 10 µM of pancracine. The numbers of proliferating cells unstained by Trypan blue were counted 24, 48, and 72 h after cell treatment with pancracine at 2.5-, 5-, 10-, and 20-μM concentrations. A (*p* ≤ 0.05) significant antiproliferative effect occurred even at the lowest tested concentration of pancracine (2.5 μM) after 48 h and 72 h of treatment for A549 and 24 h and 48 h for the MOLT-4 cell line (Figure 3A). Regarding the effect of pancracine on cell viability, a considerable difference was seen between the overall resistant adherent cell line A549 and the suspension leukemic cell line MOLT-4 of hematopoietic origin (Figure 3B). A slight decrease in viable cells occurred only at the highest concentrations tested (10 and 20 μM of pancracine) in the A549 cell line. In the case of the MOLT-4 leukemic cell line, which is particularly more sensitive to anticancer chemotherapeutic treatments, there was a significant (*p* ≤ 0.05) effect on the cell viability, beginning with the 5-μM concentration of pancracine, after a longer interval of 48 h of treatment and 10-μM pancracine after 24 h of treatment. The susceptibility of MOLT-4 and resistance of A549 cells to pancracine-induced cell death showed good correlation with the higher percentages of surviving A549 cells after the 0.25-μM doxorubicin treatment (24 and 48 h) compared to MOLT-4 cells.

### 2.2. Reduced Proliferation of A549 and MOLT-4 Cells Is Associated with Cell Cycle Redistribution

**Figure 4:**
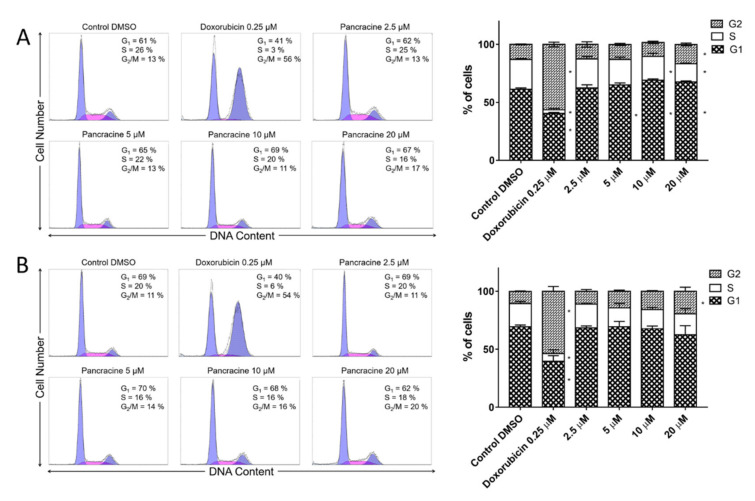
Cell cycle analysis of A549 cells after pancracine treatment. The figure shows representative histograms at 24 (**A**) and 48 (**B**) hours after treatment with 2.5-µM, 5-µM, 10-µM, and 20-µM pancracine, with a mean percentage of cells cycling through phases G1, S, and G2 from the flow cytometry measurements of three individual treatments. The bar graph summarizes the cumulative data on the percentage of cells in each phase of the cell cycle. Data are presented as the mean values ± SD, *n* = 3. * Significantly different to the control (*p* ≤ 0.05).

To determine the effect of pancracine on the cell populations at different stages of the cell cycle, a flow cytometry method was used. A significant (*p* ≤ 0.05) increase in the cell population, preferentially in the G1 phase, occurred with the use of 5-μM pancracine in A549 cells after 24 h. In addition, an increased (*p* ≤ 0.05) percentage of cells accumulated in G1, with a decrease (*p* ≤ 0.05) in S-phase cells, and a concurrent increase (*p* ≤ 0.05) in G2-phase cells occurred following the use of the highest concentration of pancracine (20 µM) and a 24-h interval of treatment (Figure 4A). A significant (*p* ≤ 0.05) increase of A549 cells in the G2 phase with a concomitant reduction of cells in the G1 and S phases was observed after 48 h treatment with 20 µM (Figure 4B).

**Figure 5:**
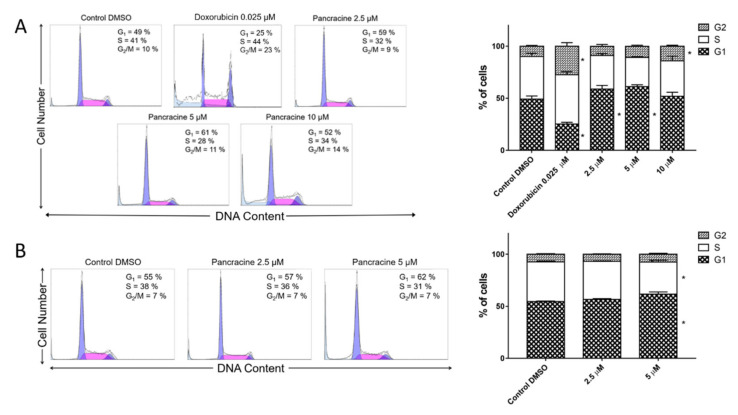
Cell cycle analysis of MOLT-4 cells after pancracine treatment. The figure shows representative histograms after (**A**) 24 h of treatment with 2.5-µM, 5-µM, and 10-µM pancracine and (**B**) 48 h of treatment with 2.5-µM and 5-µM pancracine, with a mean percentage of cells cycling through phases G1, S, and G2 from a flow cytometry measurement of three individual treatments. The bar graph summarizes the cumulative data on the percentage of cells in each phase of the cell cycle. Data are presented as the mean values ± SD, *n* = 3. * Significantly different to the control (*p* ≤ 0.05).

The situation with the MOLT-4 cell line was different. Due to its increased sensitivity leading to excessive cell death at higher doses, only lower concentrations of pancracine were used at 48 h for the cell cycle analysis of MOLT-4 cells. As shown in Figure 5A,B, a statistically significant (*p* ≤ 0.05) increase in the cell population in G1 and a decrease in the S-phase population occurred when using a 2.5-μM concentration and a 24-h treatment interval. A statistically significant (*p* ≤ 0.05) increase in the cell population in G1 and a statistically significant (*p* ≤ 0.05) decrease in the S-phase population occurred when using a 5-μM concentration and 48-h treatment. Pancracine treatment reduced the rate of cycling cells, which was accompanied by an increased fraction of sub-G1-phase cells in the DNA fluorescence histogram. In MOLT-4 cells, the application of pancracine induced an increase in the percentage of sub-G1 after 24 h (2.5 µM—5.7% ± 0.4%, 5 µM—10.2% ± 2%, and 10 µM—19.6% ± 0.7%) compared with the negative control (3.9% ± 1.9%) and an increase in the percentage of sub-G1 after 48 h (2.5 µM—5.6% ± 1.2% and 5 µM—15% ± 0.8%) compared with the negative control (4.3% ± 0.8%)—data not shown.

### 2.3. Pancracine Induced Apoptosis in MOLT-4 Cells

**Figure 6:**
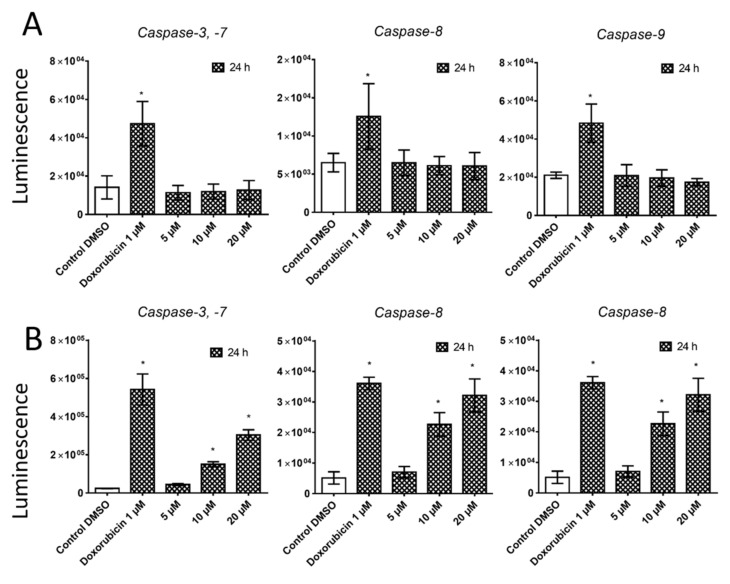
The effect of pancracine on the activity of caspase-3, capase-7, caspase-8, and caspase-9 in A549 (**A**) and MOLT-4 (**B**) cells. Results are shown as the mean ± SD from three independent experiments. * Significantly different to the control (*p* ≤ 0.05). Cells treated with 1-µM doxorubicin were used as a positive control.

**Figure 7:**
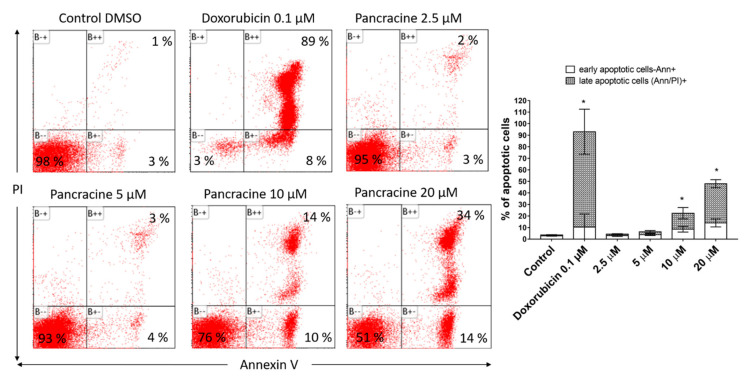
Induction of apoptosis in MOLT-4 leukemic cells after pancracine treatment. Apoptosis was determined by Annexin V and PI staining 24 h after treatment. Representative histograms of one of three independent measurements are shown. Doxorubicin at a 0.1-µM dose was used as a positive control. The bar graph represents the percentage of early and late apoptotic cells detected by flow cytometry (mean ± SD, *n* = 3). * Significantly different to the control for early and late apoptotic cells (*p* ≤ 0.05).

In order to determine whether the tumor cell death is associated with apoptosis, we used the detection of caspase-3, -7, -8, and -9 activity after 24 h of pancracine treatment in A549 and MOLT-4 cells. Each type of caspase is responsible for a specific cellular response; effector caspase-3 and -7 respond to the activation of caspase-8 for the extrinsic apoptosis pathway and to the activation of caspase-9 for activation of the intrinsic mitochondrial pathway of apoptosis. In the case of the effect of pancracine on the induction of apoptosis, no caspase activity was observed in A549 cells (Figure 6A). A clearly different situation was found after the application of 10- and 20-µM concentrations of pancracine to the MOLT-4 cell line, when there was a significant (*p* ≤ 0.05) increase in the activity of all types of caspases tested compared to the negative control (Figure 6B). Hence, to confirm further the observations that pancracine can induce apoptosis in MOLT-4, the surface exposure of phosphatidylserine and a loss in the membrane integrity was determined using Annexin and PI staining 24 h following the treatment. The combination of these fluorescent probes allowed us to separate living cells (Annexin V/PI-negative), apoptotic cells (Annexin V-positive and PI-negative), and a cell population in the late phase of apoptotic cell death (Annexin V- and PI-positive). As shown in Figure 7, 24 h after the administration of 2.5-, 5-, 10-, and 20-μM pancracine, the early apoptotic rates were 3%, 4%, 10%, and 14% and late apoptotic rates 2%, 3%, 14%, and 34%, respectively.

### 2.4. G1/S Arrest in A549 Is Associated with the Akt/p27/pRb Signaling Pathway and the Proapoptotic Activity in MOLT-4 Is Associated with Upregulation of p53 Phosphorylated at Serine 392

Based on previous results, we were interested in the detection of proteins that orchestrate cell cycle redistribution and apoptosis. To investigate the expression levels of prime upstream signaling factors and their activation—namely, cell cycle checkpoint kinase 1 (Chk1), extracellular regulated kinase 1/2 (ERK1/2), p38 mitogen-activated protein kinase (MAPK), Akt protein kinase, retinoblastoma tumor suppressor protein (pRb), p53 protein, cyclin-dependent kinase inhibitor p21 and p27, and proapoptotic protein Bax, we performed electrophoresis and Western blotting. 

**Figure 8:**
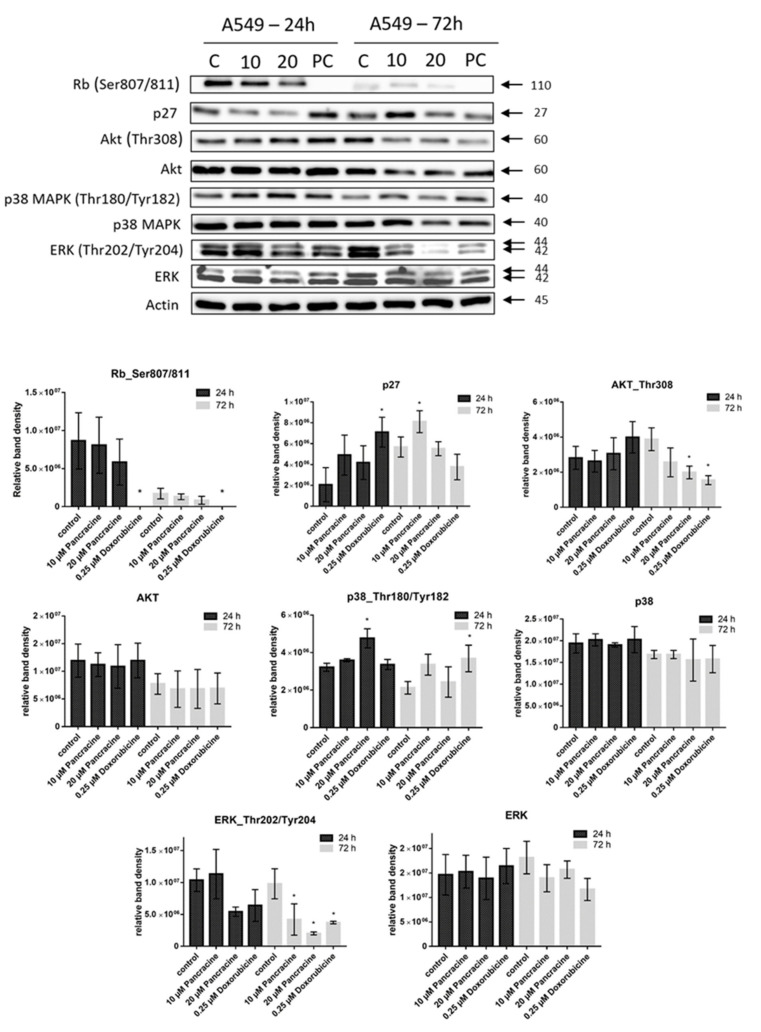
Western blot analysis of the proteins that regulate cell cycle progression, cell death, or cell survival in non-small cell lung adenocarcinoma A549 cells upon a treatment with either 10 µM or 20 µM of pancracine for 24 and 72 h. Control cells were mock-treated with 0.1% DMSO, indicated as C, and 0.25-μM doxorubicin-treated cells were used as a positive control, indicated as PC. Arrows indicate the molecular weights of proteins. The amount of selected proteins in bands was determined by densitometry. The quantitative data are shown as the relative intensity of each protein band in arbitrary units. These experiments were performed at least three times with similar results, and a cropped blot is shown from one representative experiment. Data are presented as the mean values ± SD, *n* = 3. * Significantly different to the control (*p* ≤ 0.05).

Concerning the molecular alterations in A549 cells following pancracine application (Figure 8), the antiproliferative activity was associated with significantly altered levels of various cell cycle regulatory proteins and their activated forms. The treatment with 20-µM pancracine for 24 h caused a significant (*p* ≤ 0.05) activation of p38 MAPK through phosphorylation at Thr180 and Tyr182 and decreased phosphorylation of ERK at Thr202 and Tyr204. There was neither a considerable downregulation of Akt kinase nor p27 and pRb after 24 h of treatment, whereas greater changes in the protein levels occurred after 72 h. After 72 h of exposure, pancracine dosed at 10 µM and higher decreased Akt kinase through phosphorylation on Thr308, significantly (*p* ≤ 0.05) increased the level of p27 protein, and activated the Rb protein through hypophosphorylation at Ser807/811. Furthermore, at a prolonged exposure time of 72 h, the protein levels of ERK phosphorylated on Thr202 and Tyr204 were decreased significantly (*p* ≤ 0.05) after treatment with both 10- and 20-µM pancracine, while increased the phosphorylation of p38 MAPK at Thr180 and Tyr182 shifts towards a lower 10-µM dose of pancracine. In contrast, pancracine had no effect on the levels of p53 and p21 during the entire treatment period (Supplementary Figure S1).

**Figure 9:**
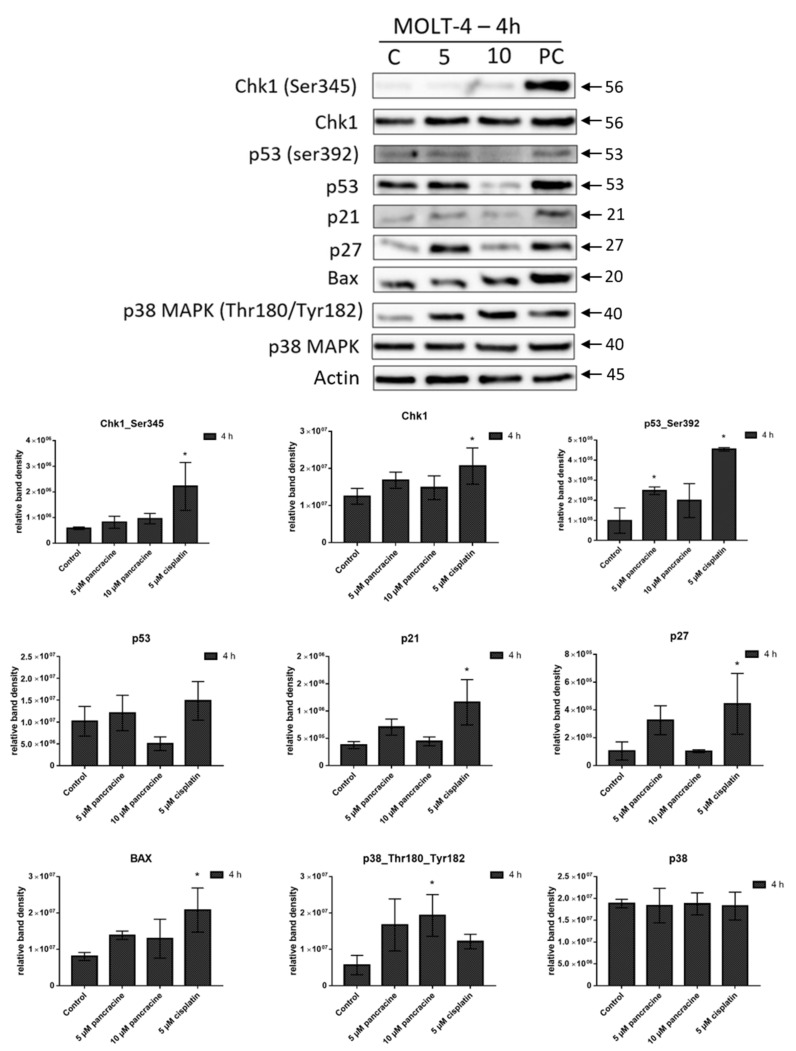
Western blot analysis of the proteins that regulate cell cycle progression, cell death, or cell survival in leukemic MOLT-4 cells upon treatment with either 5 µM or 10 µM of pancracine for 4 h. Control cells were mock-treated with 0.1% DMSO, indicated as C, and 5-µM cisplatin-treated cells were used as a positive control, indicated as PC. Arrows indicate the molecular weights of proteins. The amount of proteins in bands was determined by densitometry. The quantitative data are shown as the relative intensity of each protein band in arbitrary units. These experiments were performed at least three times with similar results, and a cropped blot is shown from one representative experiment. Data are presented as the mean values ± SD, *n* = 3. * Significantly different to the control (*p* ≤ 0.05).

To clarify the mechanism of pancracine-induced apoptosis, the expression of apoptosis-related proteins in MOLT-4 cells was determined at 4 h post-treatment using 5- and 10-µM concentrations. As shown in Figure 9, the apoptosis-inducing effect of pancracine at 5 µM was accompanied by a statistically significant (*p* ≤ 0.05) upregulation of p53 phosphorylated at Ser392. Since Ser392 phosphorylated p53 is known to be involved in the transduction of death signals, the observed increase in the amount of Bax protein correlates well. Cell cycle arrest at G1/S-phase transition occurs by the activation of inhibitors of cyclin-dependent kinases p21 and p27, which were upregulated in MOLT-4 under the same treatment (5 µM) conditions. In line with the revealed apoptosis induction, proapoptotic p38 MAPK was phosphorylated at Thr180 and Tyr182 in a dose-dependent manner, reaching significant (*p* ≤ 0.05) values with 10-µM pancracine. Reduced levels of proteins p21, p27, and p53 phosphorylated on Ser392 after 10 µM of treatment are probably due to the increased cell death of leukemic cells.

## 3. Discussion

The unique chemical structure of montanine alkaloids, with their significant biological activity, together with their rarity in nature, has led to a synthetic effort to produce ample amounts for biological studies and subsequent use in clinical therapy [15,16,17,18,19,20,21]. This also includes the preparation of montanine-type alkaloids by rearrangement of the haemantamine-type ring system [21,22]. Unfortunately, without understanding the molecular mechanisms of their actions, it is not possible to take advantage of their promising biological activity over other AAs. If we focus on the anticancer potential of montanine alkaloids, only the IC_50_ values have been available so far, with pancracine having values from 2.29 ± 0.43 µM to 5.15 ± 0.34 µM, using tumor cells derived from A549 (lung adenocarcinoma), HT-29 (colon adenocarcinoma), A2780 (ovarian carcinoma), HeLa (cervix carcinoma), MCF-7 (breast carcinoma), SAOS-2 (osteosarcoma), Jurkat (acute T-cell leukemia), and MOLT-4 (acute lymphoblastic leukemia) [6]. The IC_50_ values in the low-micromolar range were found to correspond closely to those found in an earlier study published by Cedrón et al. [9], which described the cytotoxic effect of pancracine on tumor cells with IC_50_ values ranging from 4.3 ± 0.7 µM to 9.1 ± 1.0 µM. Montanine, which is the main representative of this group of substances, seems to be more active than pancracine, because its IC_50_ values ranged from 1.04 ± 0.14 µM to 2.30 ± 0.45 µM in the study of Al Shammari et al. [23]. Since pancracine differs from montanine by only one substituent on the E ring of the 5,11-methanomorphanthridine structure, it can be hypothesized that the molecular mechanism of action of these substances may be similar. 

By selecting one apoptosis-resistant cancer cell line and one apoptosis-sensitive leukemia cell line as an experimental model counterpart, the aim of this study was to approximate the effect of pancracine on these cells, including events that lead to either cell cycle perturbation or apoptosis. 

**Figure 10:**
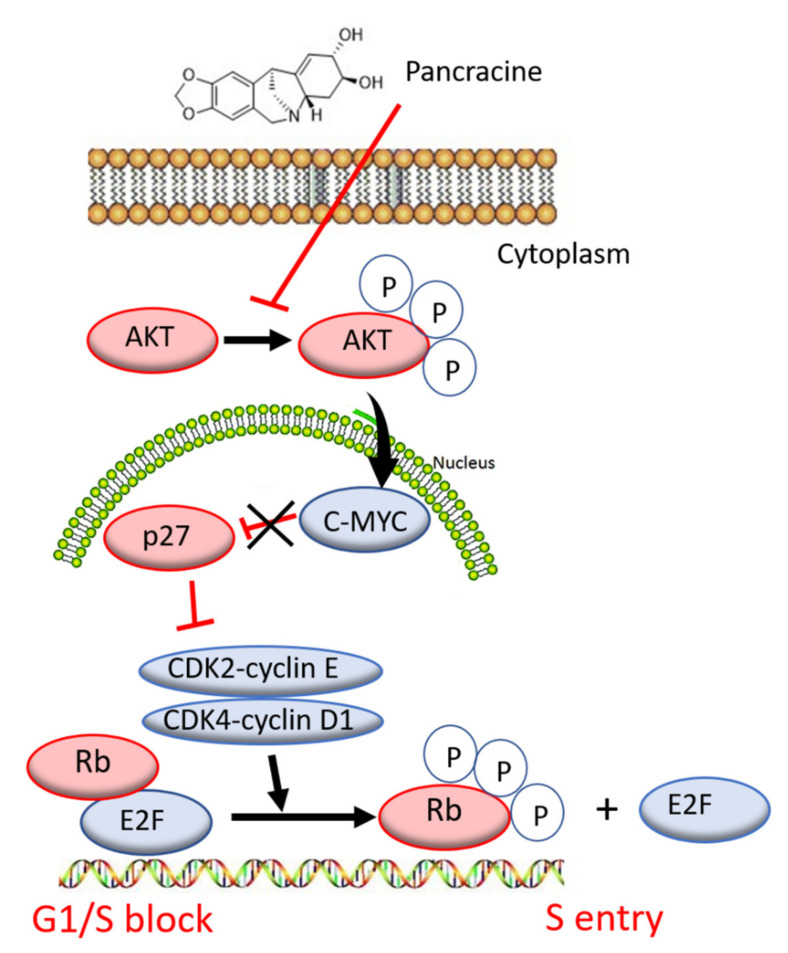
Proposed signaling pathway involved in the action of pancracine in A549 cells.

The A549 cancer cell line represents non-small cell lung cancer (NSCLC) with very poor prognosis and with high chemoresistance to the standard cytotoxic drug treatments. Considering the IC_50_ values of pancracine for A549 cells (2.29 ± 0.43 µM) [6], the strong antiproliferative effect of this compound on the A549 cancer cell line determined in this study by a real-time cell monitoring method is fully consistent with the previous findings. This growth inhibitory effect persists for 96 h. We extended this real-time cell proliferation measuring with the end-point Trypan blue exclusion test. A statistically significant antiproliferative effect on A549 cells was already demonstrated using a concentration of 2.5 mM at all time intervals (24, 48, and 72 h). The impact on cell viability of the A549 cells was negligible, as concentrations up to 20 μM failed to reduce the A549 viability below 80%. According to the real-time cell monitoring method, a 50-µM concentration of pancracine seemed to be lethal for A549 cancer cells. Cell cycle arrest at the G1/S transition occurs by the activation of several signaling pathways. One of these acts through the activation of MAPK systems by the phosphorylation of p38 MAPK, which phosphorylates several nuclear factors, such as tumor suppressor p53, activating transcription factor ATF-2, myocyte enhancer factor MEF-2, and transcription factor Myc [24]. Another possibility is signaling through the Akt kinase. The phosphorylated pAkt kinase inhibits the p27 protein [25]. p27 is a tumor suppressor that inhibits the phosphorylation of Rb by inhibition of the cyclin-dependent kinase (CDK) complex and, as a result, prevents the separation of transcription factor E2F from Rb, which, in total, prevents the transcription of genes required for G1/S transition [25]. Our results show that the proliferation of A549 cells is inhibited by cell cycle arrest in the G1 phase through the upregulation of phosphorylated p38 MAPK after 24 h and either a 10- or 20-µM pancracine treatment. Cell cycle arrest initiated through the downregulation of phosphorylated pRb protein, upregulation of p27 concomitantly with the downregulation of Akt kinase phosphorylated on Thr308, and downregulation of ERK was observed after a longer time interval (72 h) using the same experimental conditions. Besides, apoptosis is not induced at the cellular level, as proved by the activity of caspase-3/7, -8, and -9 in A549 cells. For a better overview, Figure 10 shows the schematic representation of proposed AKT/p27/pRb upstream signaling events involved in the antiproliferative activity of pancracine against A549 lung adenocarcinoma cells.

MOLT-4 is a T-lymphoblastic cell line originally derived from the peripheral blood of a 19-year-old patient with acute lymphoblastic leukemia in relapse. These cells responded to pancracine treatment with a half-maximal inhibitory concentration (IC_50_) of 2.71 ± 0.25 µM [6]. A similar strong antiproliferative effect as that seen on A549 cells was observed after the pancracine treatment of MOLT-4 cells. However, unlike in the A549 cell line, a significant strong activity in decreasing the viability of leukemic cells was observed. The viability of MOLT-4 cells decreased, even after treatment with 2.5-μM pancracine, and treatment with the higher dose of 20 μM led to a decrease of viable cells down to 20%. Due to the greater potency of pancracine with decreasing viability rates at higher concentrations, only the lower concentrations of 2.5, 5, and 10 μM after 24 h and 2.5 and 5 μM after 48 h of treatment could be used to detect the percentage of leukemic MOLT-4 cells in the cell cycle. Several publications showed that leukemic cells undergo apoptotic cell death [26,27,28]. MOLT-4 cells die due to ionizing radiation and other DNA damage-inducing agents by the apoptotic process of the activation of caspase-8 and -9 and release of cytochrome c [27]. In this study, the leukemic MOLT-4 cells died due to apoptosis activation at 5 μM, increasing the activity of effector caspase-3/7 and the activation of caspase -9 responsible for the mitochondrial apoptosis pathway and caspase-8 responsible for the pathway through death receptors. The apoptosis of MOLT-4 cells significantly increased 24 h after the exposure to a 10-μM dose of pancracine, as detected using Annexin V and PI staining. Using a concentration of 20 μM, 50% of the cells were either in the early or late phase of apoptosis. Activation of the ATM/Chk2/p53 signaling pathway was described in MOLT-4 cells after ionizing radiation exposure [29]. Additionally, the cytotoxic effect of valproic acid is accompanied by the activation of p21 and upregulation of p53 phosphorylated on Ser392 [28]. In our study, the significant apoptosis-inducing effect of pancracine was accompanied by the signaling pathway, including an increase of p27 protein, Bax, and activation of p38 MAPK through phosphorylation at Thr180 and Tyr182, and seemed to be activated by the upregulation of p53 phosphorylated at Ser392.

As far as we know, a study of the molecular mechanism of action of montanine-type AA has not yet been published, but when considering the biosynthesis of this group of alkaloids, one can consider other AA with similar structures. Haemantamine, with its α-crinane structure, also belongs to the isoquinoline group of Amaryllidaceae alkaloids, and its activity has been recently reviewed [3]. Haemantamine, even at a dose of 5 µM, also induced apoptosis in p53-deficient acute T-cell leukemia Jurkat cells [14]. In addition, alkaloids based on the montanine skeleton can be prepared by a rearrangement of the haemantamine (α-crinane)-type ring system [21,22]. The mechanism of action of haemantamine lies in the targeting of the A-site cleft on the large ribosomal subunit and rearranging rRNA to halt the elongation phase of translation, which leads to repressing cancer cell growth [30]. 

In conclusion, the later stages of preclinical testing of new chemotherapeutics have always preceded a deeper knowledge of the molecular mechanisms of their cytostatic and cytotoxic activity. Pancracine, with its strong inhibitory effect on tumor cell proliferation and viability, may be one of the potential pharmacophore scaffolds in cancer therapy. Additionally, this initial pilot study presented here attempts to open a more detailed study of the mechanisms of action of montanine-type AA.

## 4. Materials and Methods

### 4.1. Cell Culture and Culture Conditions

Experiments were performed with selected human tumor cell lines MOLT-4 (acute lymphoblastic leukemia), A549 (lung adenocarcinoma), A2780 (ovarian carcinoma), MCF-7 (breast adenocarcinoma), and HepG2 (hepatocellular carcinoma), which were purchased from the European Collection of Cell Cultures (ECACC, Salisbury, UK) and cultured in accordance with the provider’s culture method guidelines. All cell lines were maintained under standard cell culture conditions at 37 °C in a humidified incubator in an atmosphere of 5% CO_2_ and 95% air. Cells were passaged every 2 to 3 days to obtain exponential growth. Cells in the maximum range of 20 passages were used for this study.

### 4.2. Cell Treatment

Pancracine (purity > 95%) was provided by the ADINACO Research Group from the Department of Pharmaceutical Botany, Faculty of Pharmacy in Hradec Kralove, Charles University, Hradec Kralove, Czech Republic. Results of the ^1^H-NMR, HPLC/UV, and GC/MS analyses are shown in Supplementary Figures S2–S4. The compound was isolated from fresh bulbs of *Narcissus* L. cv. Professor Einstein (Amaryllidaceae) within a detailed phytochemical study [5]. Fresh stock solutions of pancracine in concentrations of 50 mM were dissolved in dimethyl sulfoxide (DMSO) (Sigma-Aldrich, St. Louis, MO, USA). Stock solutions were freshly prepared before use. For the experiments, the stock solutions were diluted with the complete culture medium to create final concentrations of 1–50 μM (50 μM is the highest concentration used in xCELLigence measurements), making sure that the concentration of DMSO was < 0.1% to avoid toxic effects on the cells. Negative control cells were sham-treated with a DMSO vehicle only (0.1%; control). Cells treated with 5% DMSO; cisplatin (Sigma-Aldrich, St. Louis, MO, USA) at 5 µM; or doxorubicin (Sigma-Aldrich, St. Louis, MO, USA) at 0.25 nM, 0.25 µM, and 1 µM were used as a positive control.

### 4.3. Screening for Antiproliferative Activity Using the xCELLigence System

The xCELLigence system (Roche, Basel, Switzerland and ACEA Biosciences, San Diego, CA, USA) was used to monitor cell adhesion, proliferation, and cytotoxicity. It was connected and tested by s resistor plate before the RTCA single-plate station was placed inside the incubator at 37 °C with an atmosphere containing 5% CO_2_. First, the seeding concentration for the experiments was optimized for each cell line. After seeding, the respective number of cells in 190 µL of medium per well of the E-plate 96 and the proliferation, attachment, and spreading of the cells were monitored every 30 min by the xCELLigence system. Approximately 24 h after seeding, when the cells were in the log growth phase, they were exposed in triplicates to 10 µL of sterile deionized water containing pancracine to obtain final concentrations of 1–50 μM. Controls received sterile deionized water + DMSO with a final concentration of 0.1%. Cells treated with 5% DMSO were used as a positive control. Growth curves were normalized to the time point of treatment. Evaluations were performed using xCELLigence 1.2.1 software (Roche, Basel, Switzerland and ACEA Biosciences, San Diego, CA, USA).

### 4.4. Proliferation and Viability Measurement Using Trypan Blue Exclusion Test

Cell proliferation and viability of MOLT-4 and A549 cells were monitored 24, 48, and 72 h after treatment with 2.5, 5, 10, and 20 μM of pancracine in the case of A549 cells (1.5 × 10^5^ cell in 5 mL placed in 25-cm^2^ Falcon flasks) and 24 and 48 h in the case of MOLT-4 cells (1 × 10^6^ cell in 5 mL placed in 25-cm^2^ Falcon flasks) with the same concentration range of pancracine. Cells treated with 0.25-µM doxorubicin were used as a positive control. Cell membrane integrity was determined using the Trypan blue exclusion technique—mixing 10 μL of 0.4% Trypan blue and 10 μL of cell suspension. The Trypan blue dye exclusion test is used to determine the percentage of viable cells and number of viable cells present in a cell suspension. It is based on the principle that live cells possess intact cell membranes that exclude certain dyes, such as Trypan Blue, whereas dead cells do not. The assay is easy to perform; a cell suspension is mixed with dye and then visually examined by microscope to determine whether cells take up or exclude the dye. Then, the individual cells are observed using a bright-field Nikon Eclipse E200 light microscope (Nikon, Tokyo, Japan); a viable cell will have a clear cytoplasm, whereas a nonviable cell will have a blue cytoplasm. In this assay, a hemocytometer (Bürker counting chamber) was used to determine exactly the number of cells in a suspension. We counted 25 squares (=1 horizontal line + 1 vertical line + 1 square). We counted only cells that were within the 0.2 mm × 0.2 mm squares. We did not count cells that overlapped over the border. The surface of each square was 0.2 mm × 0.2 mm = 0.04 mm^2^. With a height of 0.1 mm, the volume was 0.004 mm^3^. Therefore, 25 squares corresponded to a volume of 0.1 mm^3^ (=0.1 μL). Multiplication by 10,000 gave 1 mL. The calculation of proliferation in % followed this formula: (number of viable cells after treatment/number of viable cells in control DMSO) multiplied by 100. The calculation of viability in % followed this formula: (number of viable cells after treatment/number of total cells) multiplied by 100.

### 4.5. Cell Cycle Distribution and Internucleosomal DNA Fragmentation Analysis

The cells were washed with ice-cold PBS and fixed with 70% ethanol. In order to detect low molecular weight fragments of DNA, the cells were incubated for 5 min at room temperature in a buffer (192 mL of 0.2-M Na_2_HPO_4_ + 8 mL of 0.1-M citric acid, pH 7.8) and then labeled with propidium iodide in Vindelov’s solution for 1 h at 37 °C. The DNA content was determined using a CyAn flow cytometer (Beckman Coulter, Miami, FL, USA) with an excitation wavelength of 488 nm. The data were analyzed using Multicycle AV software (Phoenix Flow Systems, San Diego, CA, USA).

### 4.6. Activity of Caspases

The induction of programmed cell death was determined by monitoring the activities of caspases-3/7, caspase-8, and caspase-9 by Caspase-Glo Assays (Promega, Madison, WI, USA) 24 h after treatment with 5, 10, and 20 μM of pancracine. Cells treated with 1 µM of doxorubicin were used as a positive control. The assay provides a proluminogenic substrate in an optimized buffer system. The addition of a Caspase-Glo Reagent results in cell lysis, followed by caspase cleavage of the substrate and the generation of a luminescent signal. A total of 1 × 10^4^ cells were seeded per well using a 96-well plate format (Sigma-Aldrich, St. Louis, MO, USA). After treatment, the Caspase-Glo Assay Reagent was added to each well (50 μL/well) and incubated for 30 min before luminescence was measured using a Tecan Infinite M200 microplate reader (Tecan Group, Männedorf, Switzerland).

### 4.7. Analysis of Apoptosis

Apoptosis was determined by flow cytometry using an Alexa Fluor^®^488 Annexin V/Dead Cell Apoptosis kit (Life Technologies, Grand Island, NY, USA) in accordance with the manufacturer’s instructions. The Alexa Fluor^®^488 Annexin V/Dead Cell Apoptosis kit employs the property of Alexa Fluor^®^488 conjugated to Annexin V to bind to phosphatidylserine in the presence of Ca^2+^, and the ability of propidium iodide (PI) to enter cells with damaged cell membranes and bind to DNA. Measurements were performed immediately using a CytoFLEX LX flow cytometer (Beckman Coulter, Miami, FL, USA). List mode data were analyzed using Kaluza Analysis 2.1 software (Beckman Coulter, Miami, FL, USA).

### 4.8. Western Blot Analysis

Whole-cell lysates (Cell Lysis Buffer; Cell Signaling Technology, Danvers, MA, USA) were prepared 24 and 72 h following the treatment of A549 with 10 μM and 20 μM of pancracine and 4 h following the treatment of MOLT-4 cells with 5 μM and 10 μM of pancracine. Cells treated with 0.1% DMSO were used as a negative control. Cells treated with either 5 μM of cisplatin or 0.25 µM of doxorubicin were used as a positive control. Quantification of the protein content was performed using the BCA assay (Sigma-Aldrich, St. Louis, MO, USA). The lysates (20 µg of purified protein) were loaded into lanes of polyacrylamide gel. After electrophoresis separation, the proteins were transferred to a PVDF membrane (Bio-Rad, Hercules, CA, USA). Any nonspecific bindings of the membranes were blocked for 1 h in a Tris-buffered saline (TBS) containing 0.05% Tween 20 and 5%, *w*/*v*, nonfat dry milk. The membranes were washed in TBS. Incubation with a primary antibody against specific antigens and at the appropriate dilutions (1:1000 Chk1, 1:1000 Chk1_serine 345—Cell Signalling, Danvers, MA, USA; 1:1000 Rb, 1:1000 Rb_serine 807/811—Cell Signalling, Danvers, MA, USA; 1:10,000 β-actin—Sigma-Aldrich, St. Louis, MO, USA; 1:1000 ERK, 1:1000 ERK ½_threonine 202 and tyrosine 204—Cell Signalling, Danvers, MA, USA; 1:1000 p53 and 1:1000 p53_serine 392—Exbio, Prague, Czech Republic; 1:1000 p53_serine 15—Cell Signalling, Danvers, MA, USA; 1:1000 p21—Cell Signalling, Danvers, MA, USA; 1:1000 p27—Cell Signalling, Danvers, MA, USA; 1:1000 Akt and 1:1000 Akt_threonine 308—Cell Signalling, Danvers, MA, USA; 1:1000 p38, 1:1000 p38_ threonine 180, and tyrosine 182—Cell Signalling, Danvers, MA, USA; and 1:1000 Bax—Cell Signalling, Danvers, MA, USA) was performed at 4 °C overnight. The following day, the membranes were washed 5 times with TBS, each time for 5 min, and once with TBS for 10 min and then incubated with an appropriate secondary antibody (DakoCytomation, Glostrup, Denmark, or Cell Signalling, Danvers, MA, USA) in a dilution of 1:1000 for 1 h at room temperature. Band detection was performed using a chemiluminescence detection kit (Roche, Basel, Switzerland). To ensure equal protein loading, each membrane was reprobed, and β-actin was detected. The densities of the proteins of interest were analyzed using the GeneTools image analysis system (Syngene, Cambridge, UK).

### 4.9. Statistical Analysis

The descriptive statistics of the results were calculated and the charts made using either Microsoft Office 365 Excel (Microsoft, Redmond, WA, USA) or GraphPad Prism 7 biostatistics (GraphPad Software, La Jolla, CA, USA) software. In this study, all the values were expressed as arithmetic means with SD of triplicates unless otherwise noted. For quantitative data, normality testing was performed to assess whether parametric or nonparametric tests should be used. For experiments with parametric variables, one-way analysis of variance (ANOVA) followed by post-hoc Dunnett’s test was used to compare the mean values among different groups, with a *p*-value less than 0.05 considered as statistically significant.

## Data Availability

The descriptive statistics of the results were calculated and the charts made using either Microsoft Office 365 Excel (Microsoft, Redmond, WA, USA) or GraphPad Prism 7 biostatistics (GraphPad Software, La Jolla, CA, USA) software. In this study, all the values were expressed as arithmetic means with SD of triplicates, unless otherwise noted. For quantitative data, normality testing was performed to assess whether parametric or nonparametric tests should be used. For experiments with parametric variables, one-way analysis of variance (ANOVA), followed by post-hoc Dunnett’s test was used to compare the mean values among different groups, with a p-value less than 0.05 considered as statistically significant.

## Supplementary Materials

The following are available online at https://www.mdpi.com/article/10.3390/ijms22137014/s1.

## Abbreviations

AA	Amaryllidaceae alkaloid
BCA	bicinchoninic acid
Chk1	Checkpoint kinase 1
DMSO	dimethyl sulfoxide
ECACC	European Collection of Cell Cultures
ERK	extracellular signal-regulated kinase
IC_50_	half maximal inhibitory concentration
p53	tumor suppressor protein p53
p21	inhibitor of cyclin dependent kinase
p27	inhibitor of cyclin dependent kinase
PI	propidium iodide
PVDF	polyvinylidene difluoride
Akt	protein kinase B
RTCA	real-time cell analysis
SD	standard deviation
TBS	Tris-buffered saline
