# Supplementary material for: Pancracine, a Montanine-Type Amaryllidaceae Alkaloid, Inhibits Proliferation of A549 Lung Adenocarcinoma Cells and Induces Apoptotic Cell Death in MOLT-4 Leukemic Cells
Source: Int J Mol Sci. 2021 Jun 29;22(13):7014. doi: 10.3390/ijms22137014 (PMC8269071; doi:10.3390/ijms22137014)
Supplement: Supplementary file 1 [file ijms-22-07014-s001.zip › ijms-1246773-supplementary.pdf]

Supplementary Material for:

Pancracine, a montanine-type Amaryllidaceae alkaloid, inhibits cell growth via Akt/p27/pRb pathway and induces p53-mediated apoptosis of cancer cells

Darja Koutova[a], Radim Havelek[a*], Eva Peterova[a], Darina Muthna[a], Karel Kralovec[b], Katerina Breiterova[c], Lucie Cahlikova[c], Martina Rezacova[a]

[a]Department of Medical Biochemistry, Faculty of Medicine in Hradec Kralove, Charles University, Simkova 870, Hradec Kralove 500 03, Czech Republic

[b]Department of Biological and Biochemical Sciences, Faculty of Chemical Technology, University of Pardubice, Studentska 573, Pardubice 532 10, Czech Republic

[c]ADINACO Research Group, Department of Pharmaceutical Botany, Faculty of Pharmacy, Charles University, Heyrovskeho 1203, Hradec Kralove 500 05, Czech Republic

*Corresponding author

Radim Havelek, Ph.D.

Department of Medical Biochemistry,

Faculty of Medicine in Hradec Kralove,

Charles University,

Simkova 870,

500 03 Hradec Kralove,

Tel.: +420495816293,

E-mail address: havelekr@lfhk.cuni.cz

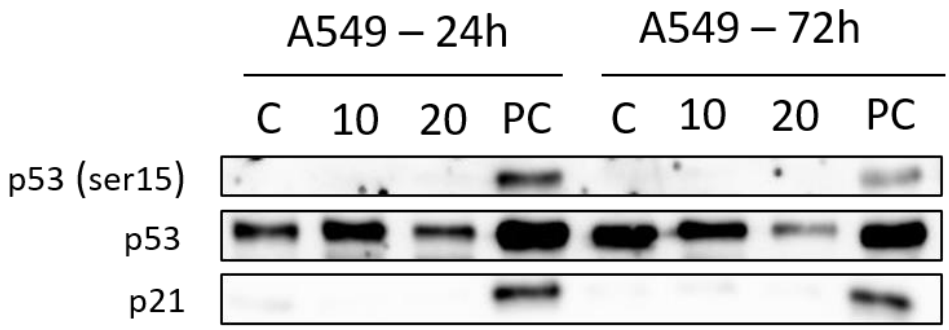

Figure S1. Western blot analysis of p53 phosphorylated at serine 15, p53 and p21 in non-small cell lung adenocarcinoma A549 cells upon treatment with either 10 µM or 20 µM pancracine for 24 and 72 hours. Control cells were mock treated with 0.1% DMSO, indicated as C, and 0.25 µM doxorubicin treated cells were used as a positive control, indicated as PC. These experiments were performed at least three times with similar results and a cropped blot is shown from one representative experiment.

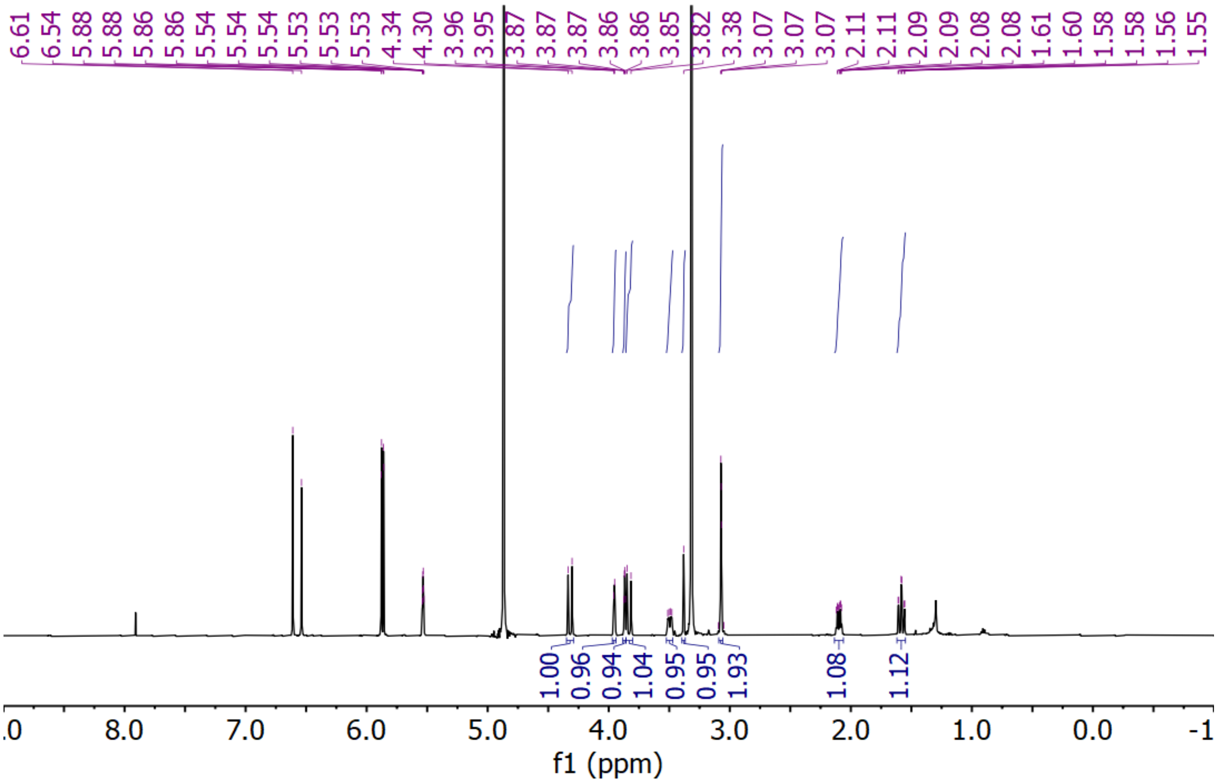

Figure S2. ^{1}H-NMR analysis of pancracine

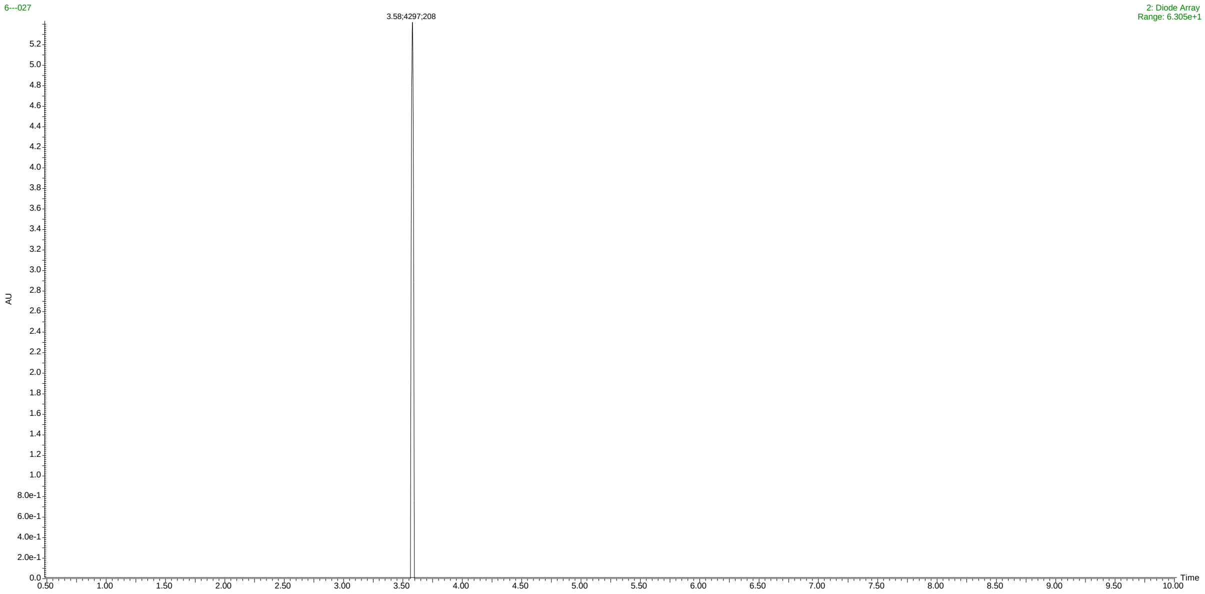

Figure S3. HPLC/UV analysis of pancracine

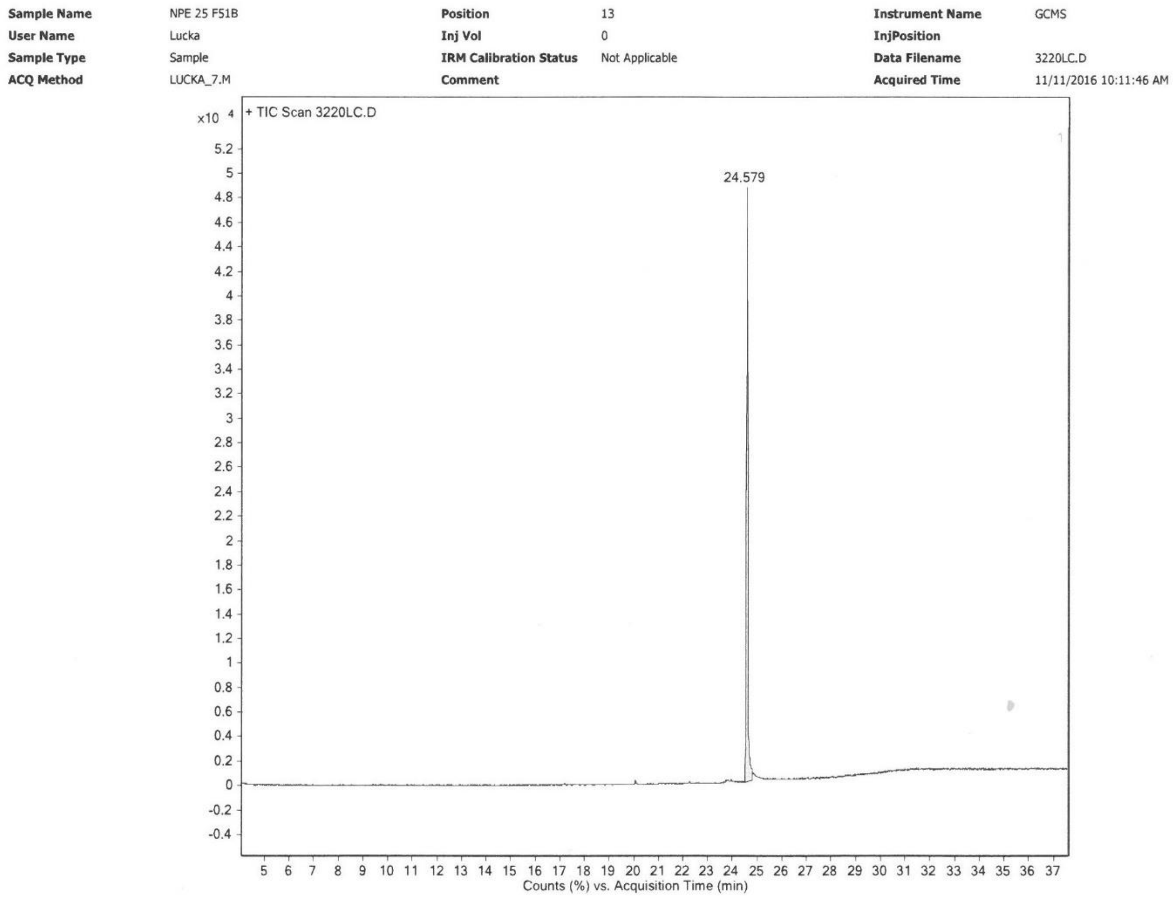

Fig. S4. GC/MS chromatogram of pancracine
